# Mechanism of Diphtheria Toxin Catalytic Domain Delivery to the Eukaryotic Cell Cytosol and the Cellular Factors that Directly Participate in the Process

**DOI:** 10.3390/toxins3030294

**Published:** 2011-03-21

**Authors:** John R. Murphy

**Affiliations:** Department of Medicine, Boston University School of Medicine, Boston, MA 02118, USA; Email: jrmrphy@bu.edu; Tel.: +1-617-638-6014; Fax: +1-617-638-6020

**Keywords:** diphtheria toxin, catalytic domain entry, coatomer complex I

## Abstract

Research on diphtheria and anthrax toxins over the past three decades has culminated in a detailed understanding of their structure function relationships (e.g., catalytic (C), transmembrane (T), and receptor binding (R) domains), as well as the identification of their eukaryotic cell surface receptor, an understanding of the molecular events leading to the receptor-mediated internalization of the toxin into an endosomal compartment, and the pH triggered conformational changes required for pore formation in the vesicle membrane. Recently, a major research effort has been focused on the development of a detailed understanding of the molecular interactions between each of these toxins and eukaryotic cell factors that play an essential role in the efficient translocation of their respective catalytic domains through the trans-endosomal vesicle membrane pore and delivery into the cell cytosol. In this review, I shall focus on recent findings that have led to a more detailed understanding of the mechanism by which the diphtheria toxin catalytic domain is delivered to the eukaryotic cell cytosol. While much work remains, it is becoming increasingly clear that the entry process is facilitated by specific interactions with a number of cellular factors in an ordered sequential fashion. In addition, since diphtheria, anthrax lethal factor and anthrax edema factor all carry multiple coatomer I complex binding motifs and COPI complex has been shown to play an essential role in entry process, it is likely that the initial steps in catalytic domain entry of these divergent toxins follow a common mechanism.

## 1. Diphtheria Toxin and Diphtheria Toxin-Based Fusion Protein Toxins

More than thirty years ago, A.M. Pappenheimer, Jr. [1] described diphtheria toxin as one of the most successfully studied of the bacterial protein toxins, and as a model system diphtheria served as a paradigm for the analysis of the other protein toxins. With recent advances in understanding the mechanism by which diphtheria toxin catalytic domain enters the eukaryotic cell cytosol, diphtheria as a model system has remained at the forefront and its study continues to provide key insights into eukaryotic cell protein::bacterial protein toxin interactions that are essential in the intoxication process.

Diphtheria toxin is synthesized by toxigenic strains of *Corynebacterium diphtheriae* in precursor form and following cleavage of its 25 amino acid signal sequence, it is released into the culture medium as a 535 amino acid single chain protein [2,3,4]. The ADP-ribosyltransferase activity of the toxin is activated by proteolytic “nicking” of the α-carbon backbone at Arg193 in an exposed 14 amino acid loop formed by a disulfide bond between Cys186 and Cys201. Upon reduction under denaturing conditions, “nicked” toxin may be separated into a 21.1 kDa *N*-terminal polypeptide (residues 1–193), which contains the catalytic (C) domain, and a 41.2 kDa *C*-terminal polypeptide (residues 194 to 535), which carries both the transmembrane (T) and receptor binding (R) domains [5,6,7,8]. The C-domain catalyzes the NAD^+^-dependent ADP-ribosylation of elongation factor 2 (EF-2), the inhibition of cellular protein synthesis and ultimately cell death by apoptosis [8,9,10]. 

Fragment B carries both the T-domain and the R-domain. The native R-domain (residues 432–535) mediates the binding of diphtheria toxin to its cell surface receptor, a heparin binding epidermal growth factor precursor (hb-EGF) [8,11]. In 1986, Murphy and co-workers pioneered the use of diphtheria toxin as a structural platform to genetically construct a family of fusion proteins toxins [12,13]. These chimeric toxins were constructed by the genetic deletion of the portion of the toxin structural gene encoding the native R-domain and its replacement with a synthetic gene encoding a surrogate receptor binding domain. The resulting fusion protein toxin is thereby directed to and selectively cytotoxic for only those target cells which express the appropriate targeted cell surface receptor. These fusion protein toxins have proved to be useful to probes in cell biology, in the study of the mechanism of toxin entry into the cytosol, and in one instance as a novel therapeutic which is now used in clinical medicine. DAB_389_IL-2 (ONTAK^®^) [14,15], was approved in 1999 by the U.S. Food and Drug Administration for the treatment of CD25 positive refractory cutaneous T cell lymphoma [16], and targets the recombinant fusion protein toxin to cells that display the high affinity IL-2 receptor.

## 2. The Intoxication Process

**Figure 1:**
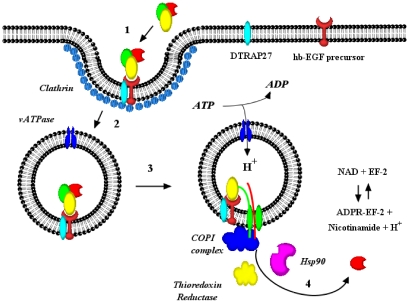
Schematic depiction of the mechanism of diphtheria toxin entry into eukaryotic cell cytosol. (1) Diphtheria toxin binds to its cell surface receptor and is (2) internalized in clathrin coated pits into early endosomal vesicles. Upon acidification of the endosomal lumen, (3) the transmembrane domain of the toxin undergoes a spontaneous dynamic reorganization and inserts into the membrane forming a pore through which (4) the C-domain is delivered to the cytosol. The delivery of the C-domain is facilitated by at least COPI complex, thioredoxin reductase and Hsp90. Once refolded into an active conformation, the C-domain catalyzes the ADP-ribosylation of elongation factor 2. Diphtheria toxin: red = catalytic domain; green = transmembrane domain; yellow = native receptor binding domain.

The intoxication of sensitive eukaryotic cells by diphtheria toxin follows an ordered series of events. As shown in Figure 1, the first step in the process is the binding of the toxin to its cell surface receptor the heparin binding epidermal growth factor-like precursor, hb-EGF [11]. This association may be enhanced by the diphtheria toxin receptor associated protein 27, DTRAP 27, which is the primate homologue of human CD9 [17]. Receptor bound toxin is concentrated in clathrin coated pits and internalized into clathrin coated vesicles (CCVs), which are then converted into early endosomal vesicles (EEVs) [18]. As the clathrin triskelon is replaced with a new set of protein components, including Arf-1 and COPI complex, the activity of the vacuolar (v)ATPase lowers the luminal pH of the EEVs. It is widely known that the acidification of the vesicle lumen triggers the dynamic unfolding of the transmembrane domain (T) [19] which allows its insertion into the endosomal vesicle membrane forming a 18–22Å pore [20,21]. Pore formation is an essential prerequisite step for the translocation of the C-domain from the *cis* (luminal) to *trans* (cytosolic) side of the EEV membrane. While debate continues over the precise mechanism and requirements for this translocation event, it is widely accepted that the formation of this cation selective membrane pore is a critical step, without which translocation of the C-domain cannot occur. We have hypothesized that the C-domain of diphtheria toxin is threaded through the pore by a process which is facilitated by a Cytosolic Translocation Factor (CTF) complex [22,23]. A second hypothesis has suggested that the nascent chaperone-like activity of the partially unfolded T-domain mediates the autonomous delivery of the C-domain across the membrane [24,25]. In either case, translocation of the C-domain is followed by reduction of the disulfide bond between Fragments A and B, which results in the release of the C-domain into the cytoplasm. Once delivered into the cytosol, the C-domain is refolded into an enzymatically active conformation and catalyzes the NAD^+^-dependent ADP-ribosylation of elongation factor 2 (EF-2), thereby inhibiting cellular protein synthesis. Upon cessation of protein synthesis the intoxicated cell will ultimately die by apoptosis [10]. In an elegant early experiment, Uchida and coworkers demonstrated that the introduction of a single molecule of fragment A is sufficient to cause the death of that cell [26].

## 3. Pore Formation, Topography and Catalytic Domain Delivery

**Figure 2:**
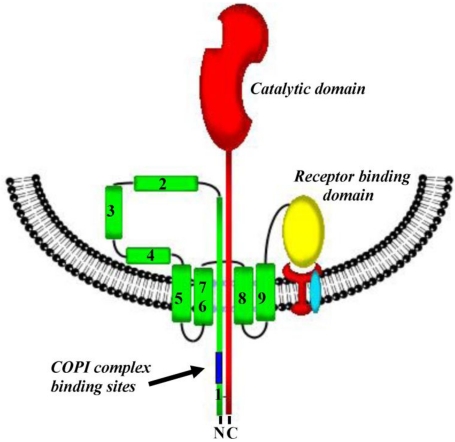
Schematic representation of the insertion of the diphtheria toxin transmembrane domain into the endosomal vesicle membrane which results in the formation of a transmembrane pore. Following furin mediated “nicking” of the toxin after Arg194 and denaturation of the catalytic domain, the *N*-terminal portion of the transmembrane domain with its disulfide bond linked *C*-terminal end of the catalytic domain appears to be threaded into the pore. Upon emergence of one or more of the KXKXX motifs on the cytosolic side of the vesicle membrane, COPI complex binds to these sequences and facilitates the translocation of the catalytic domain.

In 1976, Boquet and coworkers [19] made the critical observation that CRM45, a chain termination mutant that lacks the native R-domain, and the Fragment B in denatured diphtheria toxin had the detergent-like binding properties of integral membrane proteins. This observation led these investigators to postulate that under low pH, the T-domain of diphtheria toxin undergoes a dynamic re-organization, allowing it to insert into the vesicle membrane and provide a portal of entry into the cytosol. Donovan *et al.* [20] then demonstrated that diphtheria toxin in acidic conditions was able to form a pore in artificial lipid bilayers, a finding later extended by Kagan *et al*. [21] and Deleers *et al.* [27], who suggested that a pH gradient was required to facilitate C-domain delivery. Shiver and Donovan [28], using asolectin vesicles, demonstrated that diphtheria toxin could deliver its own C-domain across the artificial bilayer in a pH dependent fashion, independent of added proteins or factors. Interestingly, these studies demonstrated a requirement for a pH gradient, in which the endocytic vesicle luminal pH is optimally between 4.7 and 5.5 and the cytosolic pH is at or near 7.4. Once the X-ray structure of diphtheria toxin was solved, it was recognized that the acidic environment of endosomal lumen triggers the rearrangement of the T-domain of diphtheria toxin, residues 194–386, positioning the nine transmembrane helices (TH-1 through TH-9) across or adjacent to the membrane [29]. The insertion of the α-helical hairpin loop formed by TH-8 and TH-9 into the vesicle membrane is required to form a pore, and these helices may be stabilized by association with a second α-helical hairpin loop formed by TH-5 and TH-6/TH-7 [30,31]. Assays used to measure the formation and conductance of membrane pores, such as patch clamp experiments, molecular marker exclusion studies, and pH sensitive dyes have been used in conjunction with diphtheria toxin mutants to demonstrate the importance of specific residues in pore formation and support a model of helix insertion as depicted below (Figure 2).

The requirement for diphtheria toxin to pass through an acidic compartment in order to deliver its C-domain to the cytosol was established in a number of early studies, including the ability of weak bases and amines (e.g., ammonium salts, glutamine, chloroquine) [32] and ATP inhibitors to block C-domain delivery [33]. However, it was the use of specific v-ATPase inhibitors, such as Bafilomycin A-1 [34,35] that ultimately confirmed that pore forming activity was associated with the action of the v-ATPase in the endosomal vesicle membrane. 

The crystal structure of diphtheria toxin also allowed for the re-interpretation of earlier findings and the generation of toxin mutants at positions implicated in translocation. [36,37]. The T-domain (residues 195–389) is comprised of three helical layers. Layer 1 contains helices TH-1, TH-2 and TH-3, which are amphipathic in nature. Helices TH-5, TH-6, and TH-7 are hydrophobic and make up the second layer, while the third helical layer is comprised of helices TH-8 and TH-9 and forms the central core of the T-domain. One approach used to understand the topography of membrane inserted diphtheria toxin has been to examine the sensitivity of either full length diphtheria toxin or fragment B to enzymatic cleavage in cell membranes or artificial bilayers [38]. Insertion of transmembrane helices 8 and 9 is a common finding of these studies, however, discrepancies arise in interpreting cleavage products that include the C-domain and whether or not they represent translocation intermediates. For example, Madhus [39] and Madhus *et al.* [40] describe the observation of a 24–25 kDa Fragment A containing protease product derived from cell membrane bound *N*-ethylmaleimide (NEM) treated diphtheria toxin. One interpretation offered by these investigators is that this portion of the T-domain enters the cytosol and these findings suggest that sequences in TH-1 might be closely associated with C-domain residues and play a role in translocation. Further work by Madshus *et al*. [39] suggested the importance of TH-1 in translocation and implied possible interactions with an “unidentified translocation apparatus”, as will be discussed below. These early findings were prescient of recent work which has demonstrated the KXKXX COPI complex binding motifs in transmembrane helix 1 of the T-domain that are essential for catalytic domain delivery to the cytosol [41].

As early as 1981, Donovan *et al*. [20] and Kagan *et al*. [21] estimated that the pore formed by the T-domain was sufficiently large (18–22 Å) to accommodate the transit of a fully denatured C-domain from the luminal to cytosolic side of the EEV membrane. Point mutations in the T-domain of diphtheria toxin began to elucidate residues required for T-domain refolding, insertion and pore formation. Mindell *et al*. [42] and O’Keefe *et al*. [30] demonstrated the importance of residues Glu349 and Asp352 in the loop connecting TH-8 and TH-9. This loop forms a hinge which facilitates the insertion of TH-8 and TH-9 into the vesicle membrane. vanderSpek *et al*. [43] demonstrated that, while pore formation is required for C-domain delivery to the cytosol, it is not in itself sufficient. While TH-8 and TH-9 alone can create a pore [44], the pore that is formed cannot affect C-domain delivery to the cytosol. Hu *et al*. [45] demonstrated that mutations in TH-5 and TH-6 results in pore competent but non-toxic mutant forms of the toxin. Similarly, mutations in TH-1, TH-2, and TH-3 permitted the formation of intact pores, but resulted in mostly non-toxic mutants that retained full ribosyltransferase activity. Thus, detailed structural functional analysis of diphtheria toxin has characterized the cell binding, catalytic activity, and pore forming domains of diphtheria toxin, and has suggested that sequences between amino acid 194 and 280, including TH-1 through TH-4 may be involved in the translocation of the C-domain through the T-domain formed pore in the endosomal vesicle membrane.

## 4. Autonomous *vs.* Facilitated Hypotheses of Diphtheria Toxin Catalytic Domain Delivery to the Cytosol

The apparent ability of diphtheria toxin to transfer its C-domain across synthetic lipid bilayers in the absence of other proteins led to a model of autonomous C-domain delivery. London and colleagues [24] provided evidence that the diphtheria toxin T-domain, when partially inserted into membranes, can bind both its own C-domain as well as other proteins [46]. Using planar lipid membranes, Oh *et al*. [25] labeled diphtheria toxin with an *N*-terminal histidine tag (6× His) and showed that the addition of Ni^2+^ to the *trans* compartment prevented the rapid closure of pores. Based upon these observations, these investigators concluded that the 6× His-tag and presumably the amino terminal end of the C-domain are translocated from the *cis* to the *trans* side of the lipid bilayer upon pore formation by the T-domain. Ren *et al*. [24] also showed that in a low pH environment the T-domain of diphtheria converts from a shallow membrane-inserted form (capable of binding the C-domain) to a fully *trans* inserted membrane form. Thus, in the autonomous translocation model, delivery of the C-domain is achieved through the chaperone-like activity of the T-domain coincident with its full insertion into a lipid membrane. It is noteworthy that the majority of the data to support this theory is derived from studies using artificial bilayers rather than the analysis of catalytic domain translocation across the endosomal vesicle membrane. 

While diphtheria toxin may posses the ability to utilize a pH gradient in conjunction with a relatively high membrane potential to mediate a process of partial or even complete catalytic domain translocation *in vitro*, it is not at all clear that these conditions occur *in vivo*. Furthermore, numerous proteins decorate both the luminal and cytosolic face of endocytic vesicles, and a variety of membrane associated proteins are known to serve as mediators of endocytosis and vesicular trafficking, and the potential role of these factors cannot be examined with systems that employ artificial membrane bilayers.

It should be recognized that the potential requirement for cellular factors in the diphtheria toxin catalytic domain entry process was postulated as early as 1984 [47]. However, it was not until Lemichez *et al.* [48] and Ratts *et al*. [22] provided direct experimental evidence that cellular factors were essential for catalytic domain delivery to the cytosol that a hypothesis which proposed the direct participation of cellular factors in facilitating delivery was put forth. In this context, the requirement for receptor mediated endocytosis, and the findings that inhibitors of clathrin, dynamin, and (v)ATPase all block intoxication by diphtheria toxin all relate to cellular factors that are essential, but act indirectly in the C-domain entry process [18,29,49].

Ground breaking work by Lemichez *et al.* [48] on the development of an *in vitro* translocation assay using partially purified early endosomes that were pre-loaded with diphtheria toxin provided the first direct evidence to support the notion that the delivery of the C-domain to the cytosol occurs from an early endosomal compartment, and that both ATP and cellular factors were essential for this to occur. Using this *in vitro* translocation assay system, Lemichez *et al*. [36] were also the first to recognize that C-domain translocation from the lumen of endosomal vesicles to the external medium requires the participation of at least β-COP from the COPI complex.

## 5. Partial Purification and Characterization of Cytosolic Factors Required for Diphtheria Toxin Translocation

Ratts *et al*. [22] confirmed and extended the observations of Lemichez *et al*. [48] by the purification and identification of cellular factors that were essential for the *in vitro* translocation and release of the C-domain from the fusion protein toxin DAB_389_IL-2 from pre-loaded partially purified endosomal vesicles. Using the *in vitro* translocation assay developed by Lemichez *et al.* [48] as a purification assay, Ratts *et al.* [22] were able to purify a Cytosolic Translocation Factor (CTF) complex from crude Hut102 cell and yeast lysates by ca. 650-800-fold. Following mass spectrometry sequencing, the potential role of individual proteins in the translocation process was examined through the use of specific inhibitors and/or neutralizing antibodies. For example, immunodepletion of either Hsp90 or thioredoxin reductase from crude lysates of either Hut102 cells or yeast eliminated the ability of these extracts to support C-domain translocation. Whereas, the addition of recombinant Hsp90 and/or thioredoxin reductase to either of the depleted extracts was found to restore C-domain translocation activity. Finally, in combination geldanamycin and radicicol, both specific inhibitors of Hsp90, and the addition of the thioredoxin reductase stereo-specific inhibitor *cis*-13-retinoic acid to the reaction mixture were found to block C-domain translocation *in vitro*.

## 6. Interaction(s) between the Diphtheria Toxin Transmembrane Domain Helix1 and COPI Complex Proteins

**Figure 3:**
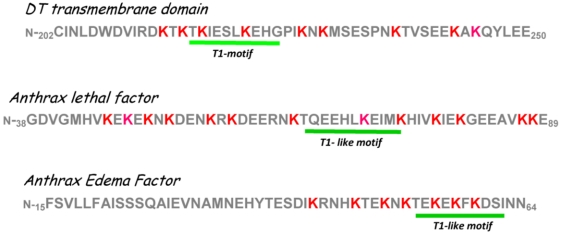
Partial amino acid sequences of *N*-terminal regions of the diphtheria toxin transmembrane domain, anthrax lethal factor, and anthrax edema factor showing the positions of their respective T1 or T1-like motifs and the clustering of multiple KXKXX COPI binding motifs on each protein.

Since the results from *in vitro* translocation experiments described above clearly suggested the direct participation of eukaryotic cell factors in the C-domain entry process, we reasoned that there might be conserved amino acid sequences in the data base whose identity could possibly lead to further insights into the C-domain entry process. Accordingly, Ratts *et al*. [23] conducted an *in silico* analysis of the primary amino acid sequence of diphtheria toxin that employed BLAST (Basic Local Alignment Search Tool) [50], and Clustal W alignment [51] analysis. In this approach overlapping 12 amino acid sequences from diphtheria toxin were used to probe the data base. Remarkably, a conserved 10 amino acid motif common to diphtheria toxin, anthrax lethal factor, anthrax edema factor and botulinum neurotoxins serotype A, C, and D was identified by the Multiple Expectation maximization for Motif Elucidation (MEME, [52]) computational search tool. This potential entry motif was designated T1, and in each instance the motif was positioned in a region of the toxin that was believed to emerge through the endosomal vesicle pore and into the cytosol early on in the entry process (Figure 3).

As described by Ratts *et al*. [23], two lines of evidence provide support for the idea that the T1 motif and adjacent amino acid residues play an essential role in the entry process. Stable transgenic Hut102 cells that express a mini-gene encoding the T1 motif corresponding to amino acids 210–229 of diphtheria toxin become >1000-fold more resistant to DAB_389_IL-2 challenge than that parental cell line. Moreover, when transgenic Hut102 cells were co-transformed with an siRNA expression vector designed to suppress the expression of the T1 motif containing peptide, the sensitivity of these cells to DAB_389_IL-2 returned to that of the parental cell line (IC_50_ = ~7 × 10^−11^ M) [23]. Based upon these observations, we reasoned that the protection afforded by T1 peptide expression was likely due to the peptide blocking an essential protein::protein interaction at a step in the C-domain entry process.

In an attempt to demonstrate such an interaction, a fusion protein was constructed between GST and a peptide that carried the T1-motif. This fusion protein was then used as bait in a series of pull down experiments with crude eukaryotic cell lysates. Both GST and the GST-DT140-271 fusion protein were used to adsorb proteins from post nuclear extracts of HUT102/6TG cells. A number of T-domain specific proteins were eluted with the GST-DT140-271 fusion protein and identified by mass spectroscopy. This analysis resulted in the identification of β-COP by immunoblot analysis [23]. Using labeled [^35^S]-β-COP that was synthesized *in vitro* using a rabbit reticulocyte transcription and translation reaction mixture, we found that GST-DT140-271 not only specifically bound and pulled down [^35^S]-β-COP, but that this interaction could be inhibited by the addition of increasing amounts of synthetic T1-motif peptide to the reaction mixture. While these observations confirmed and extended those of Lemichez *et al*. [48], they also suggested that the interactions between at least β-COP and the toxin were likely to be direct. 

It is well known that COPI is a heptameric structure that is composed of α-, β-, β'-, γ-, ε-, δ-, ζ-subunits, and the cellular functions of the complex is to facilitate endosomal vesicular trafficking and the retrograde transport of vesicles between Golgi compartments, and between the Golgi apparatus and the endoplasmic reticulum [53,54,55]. In this process, COPI complexes are recruited to the cytosolic surface of vesicle membranes *en bloc* by Arf-GTP [56,57]. Once bound to the membrane surface, secondary interactions between COPI and the cytoplasmic tails of cargo proteins and p23/24 adaptor proteins further stabilize its binding to the membrane surface. This secondary binding is mediated between dibasic signatures (KKXX, KXKXX) and/or aromatic amino acid signatures [FFXXBB(X)n] in cargo and p23/24 adaptor proteins and individual subunits of the COPI complex [58,59,60].

The presence of multiple KXKXX motifs in the T1-moif and its adjacent amino acids (Figure 3) and the emergence of these sequences on the cytosolic surface of the endosomal vesicle membrane raised the possibility that this region of the transmembrane domain could function as a mimetic of the cytoplasmic tail regions of either the cargo or p23/24 adaptor proteins and facilitate COPI binding. Trujillo *et al.* [61] tested this hypothesis by undertaking an analysis of helix 1 of the T-domain by site-directed mutagenesis and COPI binding domain swap mutagenesis. This analysis has clearly demonstrated that at least three of the four lysine residues in the region of the T1 motif are required for both COPI binding and for cytotoxic activity of DAB_389_IL-2. 

Hudson and Draper [61] previously demonstrated that at lest two pairs of closely positioned amino moieties in neomycin were required to cross-link and induce precipitation of COPI complex *in vitro*. Trujillo *et al*. [41] also described a series of experiments in which synthetic peptides whose sequences were related to transmembrane helix 1 of diphtheria toxin were used to examine COPI cross-linking and precipitation. As was previously demonstrated with neomycin, two pairs of dilysine residues in these peptides were required to induce binding and precipitation of COPI. Since the addition of the monoamine 1,3-cyclohexanebis(methylamine), CBM, to the reaction mixture blocked peptide binding to COPI complex, Trujillo *et al*. [41] reasoned that, as in the case of neomycin and the cytoplasmic tails of both cargo and p23/24 adaptor proteins, the interaction between transmembrane helix 1 and coatomer I was mediated through ε-amino moieties on the peptides. 

Perhaps, the most compelling data supporting the essential role of COPI binding to the T1 motif and adjacent lysine residues in transmembrane helix 1 in the C-domain entry process comes from the domain swap experiments reported by Trujillo *et al*. [41]. In this instance, the 13 amino acid COPI binding sequence from the cytoplasmic tail region of the p23 adaptor protein was used to replace the native T1 motif and adjacent upstream lysine residues in DAB_389_IL-2. Following it construction, expression and purification the COPI domain swap mutant DAB(212p23)_389_IL-2 was examined for cytotoxic activity against Hut102 cells *in vitro*. Dose response analysis clearly demonstrated that the domain swap mutant retained full cytotoxic potency (IC_50_ ≤ 5 × 10^−11^ M) relative to the “wild type” fusion protein toxin. These results suggest that regardless of primary sequence, the primary role for this region of the transmembrane helix 1 is COPI complex binding and that this protein::protein interaction is essential for the delivery of the C-domain to the eukaryotic cell cytosol. 

The *in silico* analysis of the diphtheria toxin by Ratts *et al*. [49] revealed the presence of T1-like motifs in anthrax lethal factor. Based upon this analysis, Tamayo *et al.* [62] used an *in vitro* translocation assay similar to that used by both Ratts *et al.* [48] and Lemichez *et al.* [47] to investigate the requirements for lethal factor delivery from the endosomal lumen to the external medium. This analysis clearly demonstrated, that like diphtheria toxin, anthrax lethal translocation requires COPI complex. Moreover, like diphtheria toxin, anthrax lethal factor must be completely denatured in the endosomal lumen before it can pass through the membrane pore formed by Protective Antigen. Furthermore, like the diphtheria toxin catalytic domain, this denaturation process was assumed to be triggered by the low pH environment in the lumen. 

In the current working model of diphtheria toxin C-domain entry (Figure 1), we envision the cytosolic delivery process to occur as follows: following binding of the toxin to its cell surface receptor and internalization into an endosomal compartment, it has been long assumed that in the acidic environment of the early endosome the catalytic domain becomes completely unfolded, it now appears as though the *C*-terminal end of the catalytic domain along with its disulfide bond linked *N*-terminal end of the transmembrane domain are treaded into the transmembrane pore. While the precise nature of the denaturation and threading remain to be fully worked out, recent preliminary observations with anthrax LFnDTA and LF suggest that cellular factors may also participate in this process as well. Nonetheless, once the multiple KXKXX sequences in transmembrane helix 1 and 2 pass through the pore and are presented on the cytosolic face of the endosomal vesicle, they appear to be recognized as either cargo and/or p23/24 cytoplasmic tail mimetics and, as such, serve as targets for COPI complex binding. It is likely that this toxin::COPI complex would be transiently stabilized on the cytosolic surface of the vesicle membrane through additional interactions between COPI complex and Arf-GTP. However, upon conversion of Arf-GTP to Arf-GDP a binary complex composed of COPI complex and the *N*-terminal portion of the T-domain would be released from the membrane surface. 

It is known that COPI complex interactions with the cytoplasmic tails of either cargo or p23/24 adaptor proteins which are anchored in the membrane effect vesicle trafficking in the cytosol. However, in marked contrast to cargo and adaptor proteins, the fully denatured catalytic domain along with its disulfide bind linked *N*-terminal portion of the transmembrane domain (e.g., transmembrane helices 1–4) are not involved in the formation of the pore. Since this portion of the transmembrane domain appears to be un-tethered, association with COPI complex may allow transmembrane helices 1–4 and the carboxy-terminal region of the catalytic domain to be “pulled” through the pore which is formed by transmembrane helices 5 through 9—much like a string may be pulled through a ring. Ratts *et al.* [48] have previously demonstrated that cytoplasmic thioredoxin reductase is essential for the translocation and release of the C-domain *in vitro*, and Hsp90 (and possibly Hsc70) most likely serve as a “refoldase” to generate its ADP-ribosyltransferase activity. It is of interest to note that Hsp90 also has been shown to be essential for the either the translocation or refolding of *Clostruidium botulinum* C2 toxin into the eukaryotic cell cytosol [62,63], the transfer of the A1 subunit of cholera toxin from the endoplasmic reticulum to the cytosol [64], and the cytosolic delivery of the anthrax related fusion protein LFnDTA [65]. 

While much work remains, it is now apparent that the molecular mechanism of diphtheria toxin catalytic domain entry into the eukaryotic cell cytosol is facilitated by target cell proteins, all acting in a systematic and ordered fashion in the delivery process. 
